# An assessment of the efficacy and safety of dydrogesterone in women with ovarian endometrioma: An open‐label multicenter clinical study

**DOI:** 10.1002/rmb2.12391

**Published:** 2021-06-04

**Authors:** Jo Kitawaki, Kaori Koga, Takumi Kanzo, Mikio Momoeda

**Affiliations:** ^1^ Department of Obstetrics and Gynecology Graduate School of Medical Science, Kyoto Prefectural University of Medicine Kyoto Japan; ^2^ Department of Obstetrics and Gynecology The University of Tokyo Tokyo Japan; ^3^ Medical Affairs Mylan EPD G.K Tokyo Japan; ^4^ Department of Integrated Women’s Health St. Luke’s International Hospital Tokyo Japan

**Keywords:** dydrogesterone, dysmenorrhea, endometriosis, ovarian endometrioma, post‐marketing study

## Abstract

**Purpose:**

To assess the efficacy and safety of dydrogesterone in Japanese women with ovarian endometrioma in a real‐world setting.

**Methods:**

The post‐marketing study involved 15 sites in Japan. Dydrogesterone 10 mg twice daily orally was administered for 21 days (day 5‐25 of each menstrual cycle) for 4 cycles. The primary outcome measure was the change in ovarian endometrioma volume from baseline. Secondary outcome measures included total dysmenorrhea score (0 = absent to 3 = severe), severity of dysmenorrhea pain [0‐10cm visual analog scale (VAS)], serum carbohydrate antigen 125 (CA‐125) levels, and safety.

**Results:**

The study group comprised women with an endometrioma aged 20 to 49 (47.4% cases aged ≥40 years). Endometrioma volume was reduced in 50% (26/52), unchanged in 25% (13/52), and increased in 25% (13/52) of women from baseline to the end of cycle 5; three‐quarters of patients thus had either reduced or unchanged ovarian endometrioma volume. Dydrogesterone significantly reduced total dysmenorrhea scores and severity of dysmenorrhea pain VAS during treatment compared with baseline. CA‐125 levels were not significantly changed during the study. The incidence of adverse events and adverse drug reactions in 59 subjects was 13.6% and 11.9%.

**Conclusions:**

Dydrogesterone prevented an increase in endometrioma size in many women, and it also significantly improved total dysmenorrhea scores and severity of dysmenorrhea pain, and was well tolerated. The ClinicalTrials.gov identifier of the study was NCT02921763.

## 1 INTRODUCTION

Endometriosis is a chronic, sex hormone‐dependent inflammatory disease characterized by the presence of endometrial tissue outside the uterine cavity, most frequently on the pelvic peritoneum and ovaries.^1^ While it is asymptomatic in some women, endometriosis is often associated with dysmenorrhea, dyspareunia, pelvic pain, and infertility.^1^ Pain is the most debilitating complaint since it has a negative impact on sexual function, social activity, ability to work, and overall well‐being and quality of life.^1^, ^2^, ^3^ Endometriosis occurs in 5‐10% of women of reproductive age, with a peak prevalence between the ages of 25 and 35 years.^1^, ^2^, ^3^, ^4^ Pharmacological therapies play a pivotal role in the long‐term management of ovarian endometriomas to control pain, and usually involve hormonal treatments aimed at suppressing ovarian function. These include combination oral contraceptives, danazol, gonadotropin‐releasing hormone (GnRH) agonists, and progestogens.^4^, ^5^ It should be noted, however, that these regimens do not improve endometriosis‐related infertility.^2^

The progestogen, dydrogesterone (Duphaston^®^), was developed, in the 1960s and shown to be effective in the relief of dysmenorrhea,^5^, ^6^, ^7^, ^8^ defined as painful menstrual cramps of uterine origin.^9^ In Japan, use of dydrogesterone for the treatment of ovarian endometriomas began in 1965; however, its use decreased after the introduction of the androgen danazol in the early 1980s, and the GnRH agonists in the late 1980s. These agents produce a pseudo‐menopausal state involving high androgen and low estrogen levels which results in atrophy of endometrial tissue.^10^, ^11^ Although danazol and GnRH agonists demonstrate efficacy in the relief of endometriosis‐associated pain,^9^, ^10^ their use may be limited by androgenic/anabolic adverse effects in the case of danazol (eg, weight gain, hot flashes, and hirsutism) and hypoestrogenic effects for GnRH agonists (eg, hot flashes, vaginal dryness, and loss of libido).^4^, ^12^, ^13^

Treatment with low‐dose estrogen/progestogen oral contraceptives (OCs) has become the first‐line pharmacological option for treating dysmenorrhea associated with endometriosis.^14^ Low‐dose or ultra‐low‐dose ethinylestradiol/norethisterone OCs^12^, ^15^ and the low‐dose ethinylestradiol/drospirenone OC,^16^, ^17^ as well as the progestogen, dienogest,^18^ have been utilized as therapies that are well tolerated and can be administered for prolonged periods of time in women with ovarian endometriomas.

There has been renewed interest in the use of progestogens for the treatment of ovarian endometriomas, due to their good tolerability, minor metabolic effects, and low cost.^19^, ^20^ However, data supporting their use is limited.^21^ As a consequence, and due to its favorable efficacy and safety profile, interest in dydrogesterone has been rekindled, more than 50 years after its first release. Dydrogesterone does not inhibit ovulation or influence basal body temperature, and it is preferable for women trying to become pregnant, and for the prevention of bleeding problems.^20^ As clinical data for Japanese patients with ovarian endometriomas treated with dydrogesterone is limited, there is a need for efficacy and safety data in this population. This post‐marketing observational study was designed to evaluate the efficacy and safety of dydrogesterone in Japanese women with ovarian endometriomas in a real‐world setting.

## 2 METHODS

This was a post‐marketing observational study conducted between June 2016 and October 2017, at 15 sites specializing in gynecology and obstetrics in Japan. The survey was conducted after being reviewed and approved by the institutional review board (IRB) and pharmaceutical affairs committee of the study site, as required. The study was conducted in compliance with the “Ministerial Ordinance Related to Standards for Conducting Post‐Marketing Surveys and Studies on Drugs” (Ordinance of the Ministry of Health, Labor and Welfare No. 171 dated December 20, 2004). The ClinicalTrials.gov identifier of the study was NCT02921763.

### 2.1 Inclusion and exclusion criteria

Women aged 20 to 49 years with an endometrioma of the ovary measuring ≥3 cm in the maximal diameter on transvaginal ultrasonography at patient enrollment, and with a menstrual cycle of 25‐38 days who were ovulating and were confirmed to have normal menstruation at patient enrollment were included in the study. All patients provided informed oral consent for participation in the study.

Main exclusion criteria were: use of gonadotropin‐releasing hormone (GnRH) agonists within 6 months prior to patient enrollment; use of hormone preparations containing progestogen and/or estrogen as an active ingredient, OCs, testosterone derivatives, or herbal products indicated for endometriosis within 3 months of patient enrollment; surgical treatment for endometriosis such as transvaginal alcohol fixation, laparotomy, or laparoscopic surgery within 2 months before patient enrollment; pregnancy, possibly pregnancy or breast‐feeding at patient enrollment; or any patient determined by the investigator/sub‐investigator to be unsuitable for other reasons.

### 2.2 Dosage and administration

Dydrogesterone (Duphaston^®^) was administered orally, 10 mg twice daily (morning and evening) for 21 days/cycle (from day 5 to day 25 of each menstrual cycle) for a total of four cycles. Thus, the total duration of the study was six cycles: 1 cycle lead‐in; four cycles treatment with dydrogesterone; and one cycle final observation period.

### 2.3 Outcome measures: Efficacy

The primary efficacy outcome was the change in volume of ovarian endometrioma from baseline to Cycle 3 and end of Cycle 5. The maximum diameter (D1) of an endometrioma on a section was measured, together with the diameter orthogonal to D1 (D2). Endometriomas were considered to be spheroid and the volume of each was calculated using the following formula: [(D1 + D2) × 1/2]^3^ × 0.52. For patients with more than one cyst, the total volume was recorded. In addition, according to changes in ovarian endometrioma volume from baseline to the end of Cycle 5, patients were divided into three groups: reduced (>−15%), unchanged (±15%), and increased (>+15%) and the number of patients in each group was counted.

Secondary outcomes were changes in the total dysmenorrhea score, severity of dysmenorrhea pain by visual analog scale (VAS), and serum carbohydrate antigen 125 (CA‐125) levels.

The severity of dysmenorrhea was scored on a 4‐point scale from 0 (absent) to 3 (severe: the patient was confined to bed for ≥1 day and was unable to work). The use of analgesics (including over‐the‐counter drugs) for the treatment of dysmenorrhea was scored on a 4‐point scale from 0 (not used) to 3 (severe: an analgesic was used for ≥3 days during the menstruation period).^11^ The total dysmenorrhea score was calculated as the sum of severity of dysmenorrhea score, and use of analgesics score^11^ and was assessed at baseline and Cycles 1 to 5 or at treatment discontinuation. A 100‐point (10 cm) VAS for dysmenorrhea was assessed at baseline and Cycles 1 to 5 or at discontinuation.

CA‐125 was measured using the standard methodology employed by each institution in everyday clinical practice at baseline and Cycle 5 or at discontinuation. The test was performed during a time of non‐menstruation.

### 2.4 Outcome measures: Safety

Safety secondary outcome measures included the incidence of adverse events (AEs) and adverse drug reactions (ADRs): an AE was defined as any unfavorable or unintended sign (including an abnormal laboratory value), symptom, or disease temporally associated with the use of a medicinal product, whether or not considered causally related to the drug. An ADR is any adverse drug event for which a causal relationship cannot be ruled out. The number of subjects with AEs, frequency, and number of AEs occurring throughout the study was summarized by System Organ Class (SOC) and Preferred Term (PT) of the MedDRA/J. AEs were recorded by type, severity, and incidence.

### 2.5 Statistical analyses

The planned sample size of the survey was 65 subjects. Categorical data were described by descriptive statistics including the mean, standard deviation (SD), median and range, and proportion of the study population. Data were analyzed statistically using Wilcoxon signed‐rank test and the non‐parametric sign test.

## 3 RESULTS

**FIGURE 1:**
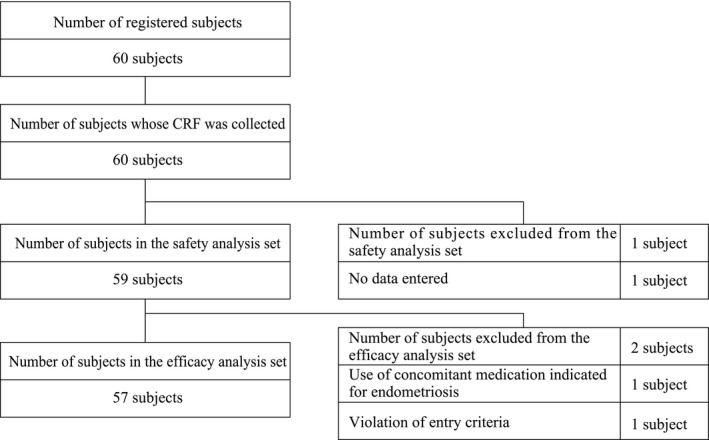
Patient disposition

Patient disposition for this study is shown in Figure 1. Of 60 subjects enrolled, 59 subjects were included in the safety analysis set, following exclusion of one subject where no data were entered. The efficacy analysis set comprised 57 subjects after exclusion of two further subjects: one for violation of entry criteria and the other for receiving concomitant medication indicated (other than dydrogesterone) for endometriosis. During the observation period, 15 subjects (25.4%) discontinued treatment. The reasons for discontinuation were onset of an AE (n = 4; 6.8%), no return to site (n = 4; 6.8%), inadequate efficacy (n = 3; 5.1%), no data obtained (n = 1; 1.7%), request for treatment with other medication (n = 1; 1.7%), request for fertility treatment (n = 1; 1.7%), and planned to undergo surgery (n = 1; 1.7%). In total, 42 women completed the study (Cycle 5).

The patient characteristics for the 57 women with endometrioma in the efficacy analysis set are shown in Table 1. Almost half the group (47.4%) were aged between 40 and 50 years, and the mean (SD) age of the cohort was 37.2 (7.1) years; the mean (SD) menstrual cycle length was 28.6 (2.4) days.

**TABLE 1:** Patient baseline characteristics (efficacy analysis set; n = 57)

Parameter		
Age (years)	Mean (SD) Median Range	37.2 (7.1) 38.0 21‐47
Age range (years)	≥20 and <30: n (%) ≥30 and <40: n (%) ≥ 40 and <50: n (%)	11 (19.3) 19 (33.3) 27 (47.4)
Height (cm)^a^	Mean (SD) Median Range	159.8 (5.0) 160.0 145.5‐170.0
Weight (kg)^a^	Mean (SD) Median Range	54.5 (9.4) 52.2 42.0‐95.0
Menstrual cycle length (d)	Mean (SD) Median Range	28.6 (2.4) 28.0 25‐38
History of allergy	Yes: n (%) No: n (%)	10 (17.5) 47 (82.5)
History of allergy to Drug : Other		1 (1.7) 9 (15.8)

^a^ n = 54.

### 3.1 Efficacy

**FIGURE 2:**
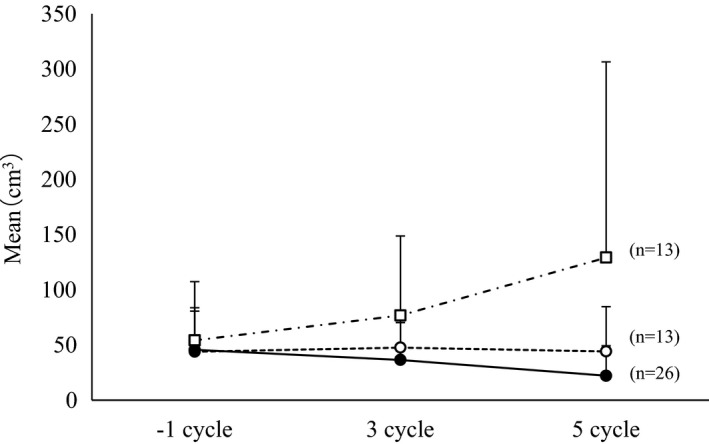
Mean (+SD) volume of ovarian endometriomas from before treatment initiation to end of Cycle 5 (efficacy analysis set) in three subgroups: endometriomal volume increased (‐⸳‐□‐⸳‐), unchanged (‐‐○‐‐), or decreased (‐●‐)

The transition of the mean (SD) volume of ovarian chocolate cysts (ovarian endometriomas) was 45.76 (40.70) cm^3^ one month before treatment, 51.97 (50.20) cm^3^ at Cycle 3 and 60.27 (109.04) cm^3^ at Cycle 5. The volume of ovarian endometriomas was reduced in 50% (26/52), unchanged in 25% (13/52), and increased in 25% (13/52) of women from baseline to the end of cycle 5. That is, three‐quarters of patients had either reduced or unchanged ovarian endometrioma volume compared with baseline by the end of cycle 5 (Figure 2).

**FIGURE 3:**
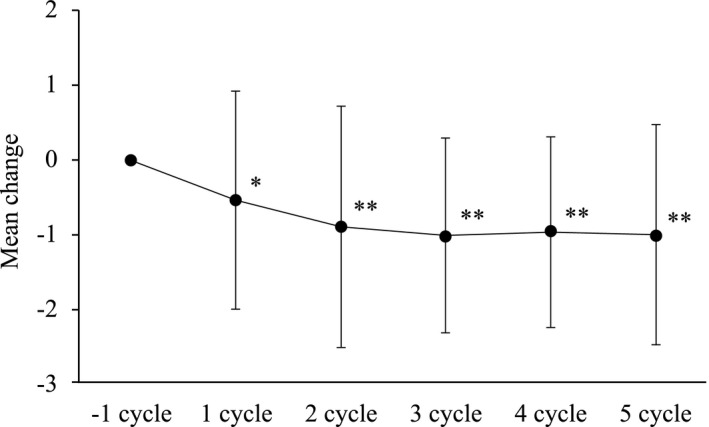
Mean change in dysmenorrhea score over time (Wilcoxon signed‐rank test (vs −1 cycle), *: *P* < .01, **: *P* < .001)

After administration of dydrogesterone, the mean total dysmenorrhea score tended to reduce over time up to Cycle 2 and remained essentially unchanged thereafter. The mean change in total dysmenorrhea score from baseline was statistically significant at Cycles 1 (*P* = .002) and Cycles 2‐5 (each *P* ≤ .0001) (Figure 3). Improvement of dysmenorrhea scores ≥1 level from baseline increased by 43.9% (25/57) at Cycle 1, 48.1% (25/52) at Cycle 2, 60.0% (30/50) at Cycle 3, 57.8% (26/45) at Cycle 4, and 57.1% (24/42) at Cycle 5. Improvement of dysmenorrhea scores ≥2 levels from baseline was 22.8% (13/57) at Cycle 1 and increased to approximately 30% at Cycles 2 to 5. Study completers (n = 42) also showed significant improvement in dysmenorrhea scores at Cycle 1 (*P* = .053), Cycle 2 (*P* = .008), and Cycles 3 to 5 (*P* < .0001). It is thought that these significant reductions in the dysmenorrhea score were caused by the fact that the severity of dysmenorrhea (score) and the use of analgesics (score), the two components of the dysmenorrhea score, both showed a tendency to improve over time. On the basis of the above, it was demonstrated that dydrogesterone in this study improved the severity of dysmenorrhea and the use of analgesics at the same time, thereby improving the dysmenorrhea score.

**FIGURE 4:**
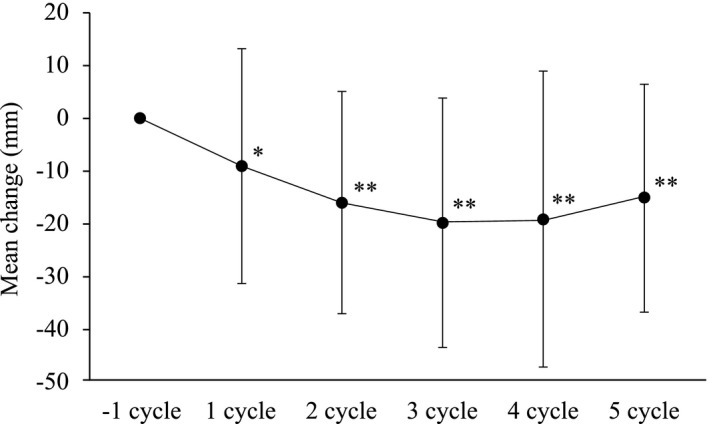
Mean change in the severity of dysmenorrhea pain (VAS) at each cycle (Wilcoxon signed‐rank test (vs −1 cycle), *: *P* < .01, **: *P* < .001)

The mean (SD) severity of dysmenorrhea pain on a VAS was reduced from 4.33 (2.92) cm at baseline to 2.01 (2.26) cm at Cycle 5 and showed a decreasing trend throughout the study. This is reflected in the mean change in the severity of dysmenorrhea pain scores (VAS) from baseline for Cycle 1 (*P* = .0024) and Cycles 2, 3, 4, and 5 (*P* < .0001 for each) (Figure 4). The percentage change of dysmenorrhea pain (VAS) was also significantly reduced at all these time points compared with baseline (Figure 4). Study completers (n = 42) also achieved significant improvement in mean severity of dysmenorrhea pain (VAS) from Cycles 2 to 5 (*P* ≤ .0001), and mean percentage change of severity of dysmenorrhea pain (VAS) at Cycle 2 (*P* = .0009), Cycle 3 (*P* = .001), Cycle 4 (*P* = .0002), and Cycle 5 (*P* = .0003).

The mean (SD) change in serum CA‐125 levels from baseline to Cycle 5 was −6.47 (97.38) U/mL and was not statistically significant (*P* = .804). Similarly, the mean (SD) percentage change of CA‐125 levels was 38.00 (183.02) % and was not significant (*P* = .819).

### 3.2 Safety

The incidence of AEs and ADRs in the study was 13.6% (8/59 subjects) and 11.9% (7/59 subjects), respectively. The most common AE was abnormal uterine bleeding (5.1%; 3/59 subjects). AEs were mainly mild (9 events in 6 subjects) or moderate (1 event in 1 subject). One serious AE of breast cancer for which the causality was not deniable was observed in one subject (1.7%). AEs were most frequently observed in Cycle 3 (5 events in 5 subjects), followed by Cycle 2 (4 events in 3 subjects) and Cycles 1 and 5 (both 1 event in 1 subject). AEs that led to treatment interruption and discontinuation occurred in 4/59 (6.8%) subjects: these were all single events (abnormal uterine bleeding and irregular menstruation, breast cancer, constipation, and somnolence).

All AEs and ADRs resolved or improved. Abnormal changes in laboratory test results were not observed in any of the 59 subjects in the safety analysis set.

## 4 DISCUSSION

In this real‐world observational study of endometriosis patients with ovarian endometriomas, dydrogesterone 10 mg twice daily for 21 days (on days 5‐25 of the menstrual cycle) significantly improved total dysmenorrhea scores (severity of symptoms plus use of rescue analgesics) and severity of dysmenorrhea pain symptoms (VAS), and it was well tolerated. Furthermore, three‐quarters of patients had either reduced or unchanged endometrioma volume. Serum CA‐125 levels, a well‐established marker for endometriosis,^22^ were slightly reduced by dydrogesterone at the end of Cycle 5, although the difference was not statistically significant. These findings are consistent with an early study which found that dydrogesterone administered during the luteal phase significantly reduced pain, but did not improve laparoscopic appearance.^23^ Similarly, a review of older data also indicated that dydrogesterone alleviated pain in many patients with endometriosis and/or dysmenorrhea.^20^ Findings from early uncontrolled studies showed that many women with endometriosis became symptom‐free or experienced a significant reduction in the occurrence and severity of symptoms when treated with dydrogesterone.^24^, ^25^, ^26^, ^27^, ^28^ Regression of lesions was also reported, although the extent to which these reduced in size or disappeared differed across the studies. The current post‐marketing observational study used transvaginal ultrasonography to measure the size of ovarian endometrioma, and thus, cannot be compared to the early studies that investigated lesions laparoscopically, and in three‐quarters of those treated with dydrogesterone (39/52) no enlargement was demonstrated.

A recent open‐label trial of Japanese patients with dysmenorrhea reported that dydrogesterone significantly reduced total dysmenorrhea VAS scores and dysmenorrhea‐related pain symptoms.^29^ In agreement with the present study, dydrogesterone was well tolerated although the trial reported a higher incidence of ADRs than this survey (31.8% versus 11.9%) and a higher incidence of the most common AE—abnormal uterine bleeding (29.5%; versus 5.1%).^29^ These findings possibly reflect differences in the methodology for recording AEs, since in the Taniguchi study each patient recorded such events in a diary which is a more active method than used in the current study (survey and recorded on case report forms). In addition, different dosages were employed in the two studies (10 mg/d in the Taniguchi study vs 20 mg/d in our study) and this may explain some AEs such as abnormal uterine bleeding, although the mechanisms involved are not well understood.

This real‐world observational study has a number of limitations which need to be acknowledged. Firstly, it did not include a control group and so all conclusions about the relative effectiveness and safety of dydrogesterone must be guarded. Comparisons with baseline (prior to treatment) may be influenced by changes related to time. Finally, while the number of women included in the study seemed reasonable when it was started, for the comparison of endometrioma sizes it was likely small as the standard deviations were very large and made it difficult to identify any statistically significant differences.

Endometriosis is a chronic and often recurrent condition, and long‐term treatment is usually required. Progestins such as dydrogesterone have their place today in the symptomatic management of pain and other symptoms caused by endometriosis, especially when long‐term treatment is required.^19^ Dydrogesterone is preferable in cases where the individual is trying to become pregnant and to prevent bleeding problems, since it can be used cyclically, has no androgenic side effects and ovulation is not inhibited.^19^ Given the relative paucity of data with dydrogesterone in women diagnosed with endometriosis, our findings are useful to gynecologists since they highlight its potential value in this clinical setting. Pain management is a key component of the care process for women with endometriosis who may also be experiencing debilitating symptoms such as dysmenorrhea, abdominal discomfort, and/or dyspareunia which can have a marked negative impact on their quality of life. The drug was well tolerated, and no new safety signals were recorded.

In conclusion, this post‐marketing study in everyday clinical practice adds to the limited data concerning the efficacy and safety of dydrogesterone for the long‐term treatment of Japanese women with endometriosis. For the majority of those treated (75%), dydrogesterone prevented an increase in endometrioma size. It also significantly improved total dysmenorrhea scores and severity of dysmenorrhea pain, and was well tolerated. These results indicate that it may be a suitable long‐term treatment option for patients who are symptomatic, including those who had previously undergone surgery.

## CONFLICTS OF INTEREST

Takumi Kanzo is an employee of Mylan EPD G.K, Tokyo, Japan. The other authors have no conflicts of interest to disclose related to this work.

## ETHICAL APPROVAL

This study was conducted after being reviewed and approved by the institutional review board (IRB)/ethics committee and pharmaceutical affairs committee of the study site, as required. The study was also conducted in compliance with the “Ministerial Ordinance Related to Standards for Conducting Post‐Marketing Surveys and Studies on Drugs” (Ordinance of the Ministry of Health, Labor and Welfare No. 171 dated December 20, 2004).

## References

[1] Zondervan KT , Becker CM , Koga K , Missmer SA , Taylor RN , Viganò P . Endometriosis. Nat Rev Dis Primers. 2018;4:9. 10.1038/s41572-018-0008-5 30026507

[2] Ferrero S , Evangelisti G , Barra F . Current and emerging treatment options for endometriosis. Expert Opin Pharmacother. 2018;19:1109‐1125. 29975553 10.1080/14656566.2018.1494154

[3] Camara O , Herrmann J , Egbe A , et al. Treatment of endometriosis of uterosacral ligament and rectum through the vagina: description of a modified technique. Hum Reprod. 2009;24:1407‐1413. 19223289 10.1093/humrep/dep016

[4] Crosignani P , Olive D , Bergqvist A , Luciano A . Advances in the management of endometriosis: an update for clinicians. Hum Reprod Update. 2006;12:179‐189. 16280355 10.1093/humupd/dmi049

[5] Vercellini P , Viganò P , Somigliana E , Fedele L . Endometriosis: pathogenesis and treatment. Nat Rev Endocrinol. 2014;10:261‐275. 24366116 10.1038/nrendo.2013.255

[6] Bishop PM . A new oral progestogen in the treatment of dysmenorrhoea. Proc R Soc Med. 1961;54:752‐754. 13869503 10.1177/003591576105400916 PMC1870545

[7] Bell ET , Loraine JA . Effect of dydrogesterone on hormone excretion in patients with dysmenorrhoea. Lancet. 1965;1:403‐406. 14238092 10.1016/s0140-6736(65)90003-6

[8] Fairweather DV . Duphaston in dysmenorrhoea; the results of a double blind clinical trial. J Obstet Gynaecol Br Commonw. 1965;72:193‐195. 14273095 10.1111/j.1471-0528.1965.tb01416.x

[9] Iacovides S , Avidon I , Baker FC . What we know about primary dysmenorrhea today: a critical review. Hum Reprod Update. 2015;21:762‐778. 26346058 10.1093/humupd/dmv039

[10] Farquhar C , Prentice A , Singla A , Selak V . Danazol for pelvic pain associated with endometriosis. Cochrane Database Syst Rev. 2007;(4):CD000068. 10.1002/14651858.CD000068.pub2 PMC12740266 17943735

[11] Brown J , Pan A , Hart RJ . Gonadotrophin‐releasing hormone analogues for pain associated with endometriosis. Cochrane Database Syst Rev. 2010;(12):CD008475. 10.1002/14651858.CD008475.pub2 PMC7388859 21154398

[12] Harada T , Momoeda M , Taketani Y , Hoshiai H , Terakawa N . Low‐dose oral contraceptive pill for dysmenorrhea associated with endometriosis: a placebo‐controlled, double‐blind, randomized trial. Fertil Steril. 2008;90:1583‐1588. 18164001 10.1016/j.fertnstert.2007.08.051

[13] Brown J , Farquhar C . An overview of treatments for endometriosis. JAMA. 2015;313:296‐297. 25603001 10.1001/jama.2014.17119

[14] Harada T . Dysmenorrhea and endometriosis in young women. Yonago Acta Med. 2013;56:81‐84. 24574576 PMC3935015

[15] Harada T , Momoeda M . Evaluation of an ultra‐low‐dose oral contraceptive for dysmenorrhea: a placebo‐controlled, double blind, randomized trial. Fertil Steril. 2016;106:1807‐1814. 27717552 10.1016/j.fertnstert.2016.08.051

[16] Mabrouk M , Solfrini S , Frascà C , et al. A new oral contraceptive regimen for endometriosis management: preliminary experience with 24/4‐day drospirenone/ethinylestradiol 3 mg/20 mcg. Gynecol Endocrinol. 2012;28:451‐454. 22132832 10.3109/09513590.2011.634936

[17] Harada T , Kosaka S , Elliesen J , Yasuda M , Ito M , Momoeda M . Ethinylestradiol 20 μg/drospirenone 3 mg in a flexible extended regimen for the management of endometriosis‐associated pelvic pain: a randomized controlled trial. Fertil Steril. 2017;108:798‐805. 28911925 10.1016/j.fertnstert.2017.07.1165

[18] Schindler AE . Dienogest in long‐term treatment of endometriosis. Int J Womens Health. 2011;3:175‐184. 21792339 10.2147/IJWH.S5633 PMC3140813

[19] Vercellini P , Fedele L , Pietropaplo G , Frontino G , Somigliana E , Crosignani PG . Progestogens for endometriosis: forward to the past. Hum Reprod Update. 2003;9:387‐396. 12926531 10.1093/humupd/dmg030

[20] Schweppe KW . The place of dydrogesterone in the treatment of endometriosis and adenomyosis. Maturitas. 2009;65(Suppl 1):S23‐S27. 19945806 10.1016/j.maturitas.2009.11.011

[21] Brown J , Kives S , Akhtar M . Progestagens and anti‐progestagens for pain associated with endometriosis. Cochrane Database Syst Rev. 2012;(3):CD002122. 10.1002/14651858.CD002122.pub2 PMC6885053 22419284

[22] Chen FP , Soong YK , Lee N , et al. The use of serum CA‐125 as a marker for endometriosis in patients with dysmenorrhea for monitoring therapy and for recurrence of endometriosis. Acta Obstet Gynecol Scand. 1998;77:665‐670. 9688246 10.1034/j.1600-0412.1998.770615.x

[23] Overton CE , Lindsay PC , Balroop Johal MB , et al. A randomized, double‐blind, placebo‐controlled study of luteal phase dydrogesterone (Duphaston) in women with minimal to mild endometriosis. Fertil Steril. 1994;62:701‐707. 7926076 10.1016/s0015-0282(16)56991-x

[24] Johnston WIH . Dydrogesterone and endometriosis. Br J Obstet Gynaec. 1976;83:77‐80. 10.1111/j.1471-0528.1976.tb00734.x 1252380

[25] Kaiser E , Wagner THA . Die Behandlung der Endometriose mit Dydrogesteron. TW Gynaekologie. 1989;2:386‐388.

[26] Walker SM . The treatment of endometriosis with dydrogesterone. Br J Clin Pract. 1983;S24:40‐46.

[27] Tumasian KP , Bēspoyasnaya VV , Voronovskaya IV . Treatment of endometriosis in female infertility. Lik Sprava. 2001;3:103‐105. 11559992

[28] Cornillie FJ , Puttemans P , Brosens IA . Histology and ultrastructure of human endometriotic tissues treated with dydrogesterone (Duphaston). Eur J Obstet Gynecol Reprod Biol. 1987;26:39‐55. 3666263 10.1016/0028-2243(87)90008-6

[29] Taniguchi F , Ota I , Iba Y , et al. The efficacy and safety of dydrogesterone for treatment of dysmenorrhea: An open‐label multicenter clinical study. J Obstet Gynaecol Res. 2018;45:168‐175. 30246276 10.1111/jog.13807

